# Geometric Affordance Perception: Leveraging Deep 3D Saliency With the Interaction Tensor

**DOI:** 10.3389/fnbot.2020.00045

**Published:** 2020-07-07

**Authors:** Eduardo Ruiz, Walterio Mayol-Cuevas

**Affiliations:** Visual Information Lab, Department of Computer Science, University of Bristol, Bristol, United Kingdom

**Keywords:** affordance, affordance detection, visual perception, learning, cognitive robotics

## Abstract

Agents that need to act on their surroundings can significantly benefit from the perception of their interaction possibilities or affordances. In this paper we combine the benefits of the Interaction Tensor, a straight-forward geometrical representation that captures multiple object-scene interactions, with deep learning saliency for fast parsing of affordances in the environment. Our approach works with visually perceived 3D pointclouds and enables to query a 3D scene for locations that support affordances such as sitting or riding, as well as interactions for everyday objects like the where to hang an umbrella or place a mug. Crucially, the nature of the interaction description exhibits one-shot generalization. Experiments with numerous synthetic and real RGB-D scenes and validated by human subjects, show that the representation enables the prediction of affordance candidate locations in novel environments from a single training example. The approach also allows for a highly parallelizable, multiple-affordance representation, and works at fast rates. The combination of the deep neural network that learns to estimate scene saliency with the one-shot geometric representation aligns well with the expectation that computational models for affordance estimation should be perceptually direct and economical.

## 1. Introduction

The concept of affordance was coined by Gibson (1979) more than five decades ago in the field of ecological psychology. For Gibson, affordances are action opportunities in the environment that are *directly* perceived by the observer. According to this, the goal of vision was to recognize the affordances rather than independent elements or objects in the scene. In this sense, the visual perception problem becomes that of recovering the *invariant* properties “offered” by the environment.

Being un-linked from action and perhaps motivated by the top-down view adopted in computer vision research, much of the attention given to the problem of affordances has focused on the recovery of complex representations of the world, internal symbolic relationships or semantic category information, which undermines the very notion of *direct* and economical perception of affordances.

The idea of being able to directly determine affordances has faced many dilemmas, namely the challenging problems of visually recovering the relevant properties of the environment in a robust and accurate manner. These problems are further accentuated in robotics, due to the fact that robots need to be able to work in environments that are cluttered, unstructured, and previously unknown. Developing a method that is able to work under these conditions is a difficult problem. More so when traditional affordance detection approaches often need to recognize objects semantically in the environment, or they need to have extensively trained for as many cases (examples) as possible in order to generalize to novel scenarios. Robots would benefit from affordance detection approaches that do not rely on object recognition, nor environment's features costly to estimate; dropping or relaxing such requirements in the perception system can allow robots to have greater generalization capabilities enabling them to accomplish their task efficiently.

We argue that in order to truly perceive affordances in the environment in a way that is most useful for cognitive robots, there is a need for methods that are agnostic to object categories and free from complex feature representations; paraphrasing Gibson, to perceive an affordance is not to classify an object (Gibson, 1979). Furthermore, affordance detection methods for robotic systems need to allow for fast computations and be able to generalize to novel scenarios without lengthy and costly training phases. We hypothesize that geometry on its own provides enough information to robustly and generically characterize affordances in such ways.

**Figure 1:**
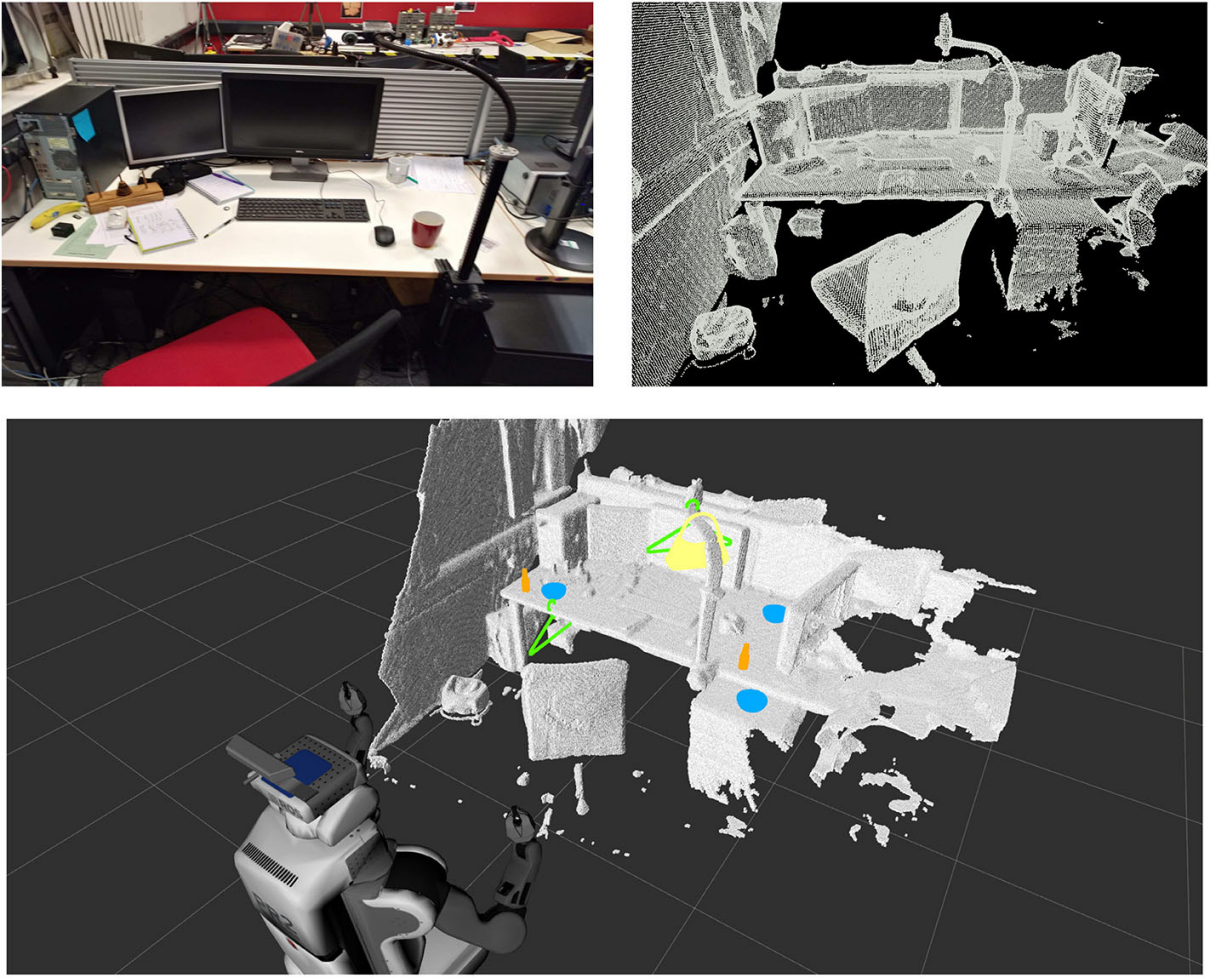
Our affordance perception approach enables to go into a completely unknown environment and predict good candidate locations affording to Place, Hang, Fill, etc. everyday objects and also *human* affordances such as Sitting. With the proposed approach we are able to determine over 80 affordances simultaneously in real-time on never before seen RGB-D scenes and from a single interaction example. These results are achieved without a script of what goes where or any prior scene knowledge yet score highly as judged by human observers.

In previous work (Ruiz and Mayol-Cuevas, 2018), we introduced a method to characterize interactions between a 3D scene and an object of interest (e.g., pointclouds from synthetic models), which allows for affordance prediction in visually perceived environments (e.g., pointclouds from RGB-D sensors): The Interaction Tensor. In this paper, we extend our work to allow for several dozens of affordances to be predicted simultaneously in real-time in unknown scenarios, training from a single example per interaction. Furthermore, we present a method that combines the rich geometric information of the Interaction Tensor with saliency detection learned by a deep-learning architecture. Figure 1 shows examples of the results of our proposed method in a previously unknown RGB-D scene.

This paper is organized as follows. Section 2 presents a review of the related previous works in affordance perception and learning. Section 3 presented the core of our proposed affordance representation and the one-shot learning algorithm to detect affordances in novel (i.e., unknown) synthetic and real RGB-D scans of indoor environments. Section 4 introduces the hybrid approach that allows to devise an optimized multiple-affordance representation by leveraging a state-of-the-art deep learning method to efficiently parse the 3D input. Section 5 presents our experiments and evaluations. Finally, section 6 contains the conclusions and final discussion of our work.

## 2. Affordance Learning and Perception

Works considering affordance perception appeared in the computer vision and robotics communities during the early 1990s decade (e.g., Ballard and Brown, 1992; Duchon et al., 1998). Since then, these fields have given an important amount of attention to the study of affordances. In this section we first review previous approaches for affordance perception and learning; then, we summarize them and introduce the motivation for our method. In this section we also outline the contributions of our work in contrast with existing approaches.

### 2.1. The Computation of Affordances

As reviewed by Horton et al. (2012) works that incorporated ideas from affordance perception and learning appeared early on in robotics literature, even before affordances became a popular topic in the area. Robotic systems with *simple* yet efficient perception systems were tightly coupled to mechanisms for planning and acting (Brooks, 1986), works that took inspiration from ideas of reactive or behavior-based robotics and developmental robotics. In fact, a large body of research in affordances for robotic systems comes from the developmental robotics field (Asada et al., 2009; Cangelosi and Schlesinger, 2015; Min et al., 2016; Jamone et al., 2018). The main idea of many of these approaches is to learn a mapping between percepts and actions by observing the consequences of robot actions in the environment. Early work of Montesano et al. (2007, 2008) studied the problem of affordance learning as a problem of structure learning, representing affordances as the (probabilistic) relations between actions, objects, and effects. With such a model a robot is able to learn a mapping between basic exploratory actions, such as tap and grasp, and the effects that those actions have on an object. Later works have built on Montesano's model and have used statistical relational learning to encode relations between (afforded) actions and *percepts*. For instance, extending the model to encode the effects of single-object actions relative to other objects, which allows the robot to learn two-object *relational* affordances (Moldovan et al., 2012), higher-level manipulation actions such as *makeSpace* or *moveAround* (Moldovan et al., 2013), and two-arm manipulation (Moldovan and De Raedt, 2014a). Furthermore, with a similar model, Moldovan and De Raedt (2014b) learn co-occurrence probabilities for occluded object search using a list of object properties and afforded-actions.

The study of *relational* affordances or the effect that actions on an object cause on another have also been studied in order to learn tool usage. Seminal work of Stoytchev (2005) showed that affordance representations can be used to solve tool-using tasks by dynamically sequencing exploratory behaviors. More relevant examples of such approaches are the works by Sinapov and Stoytchev (2007), Tikhanoff et al. (2013), Goncalves et al. (2014), Dehban et al. (2016), Antunes et al. (2016), and Saponaro et al. (2017), where the focus is on learning a mapping that allows to plan and achieve a target effect or object configuration in a table-top manipulation scenario. More complex feature combinations (Mar et al., 2015) and more tool options (Mar et al., 2017) are considered by Mar and colleagues, who additionally take into account the way in which the tools are grasped (e.g., rotation around handle). Following a similar approach, based on exploration, works have used low-level learned behaviors to bootstrap *complex* affordance learning (e.g., *stackability*). Examples of these methods are Ugur et al. (2014, 2015), Ugur and Piater (2014, 2015, 2017), where a robot learns rules and object-effect categories such as *unstable, hollow, solid, inserted*, etc. that allow him to build a plan in a tower-building task.

Another important amount of research has been dedicated to the detection and learning of *grasping* affordances, which is a key skill for robots that need to interact with the world. A good example of works in this area is Kroemer et al. (2012), who presented a method to directly link object parts to motor priors for manipulation. In a similar fashion, methods have been proposed to learn grasp densities capable to generalize over various partly-similar objects (Detry et al., 2009, 2012, 2013) or generate grasp hypotheses for pile clearing tasks in order to perform the grasp with the lowest risk of disturbing the pile (Katz et al., 2014). In Song et al. (2013, 2015), an approach that leverages Bayesian Networks is used to learn task-constrained grasp. The work presented learns grasps that take into account robot embodiment, task requirements, and is able to generalize based on human demonstrations. More recently, *enveloping* grasp affordances and antipodal grasps were studied in by Pas and Platt (2016, 2018). These approaches build on geometrical shapes fitted to pointcloud regions to generate grasp hypotheses. By using curvature, normals and quadric surfaces fitted to pointcloud patches, and then HOG features on pointcloud 2D projections, a robot is able to grasp objects in cluttered scenarios with high rates of success.

Focusing on learning what the environment affords to others, e.g., humans, by observing them performing a task or interacting with objects has also been widely studied. Typical examples here can be found in Human-Robot Interaction research; for instance, robots that learn to recognize gestures and anticipate human actions (Saponaro et al., 2013; Jiang and Saxena, 2014). In Chan et al. (2014), a robot learns proper grasp configurations for object handover by observing humans using tools such as knives and screwdrivers, in Shu et al. (2017), a robot learns *social* affordances e.g., human-like behaviors, in human-robot interaction scenarios such as *waving, shaking hands*.

In contrast with the methods presented so far, where the system (e.g., robot) learns affordances by *observing* the effects of the interactions, studies in affordances have also made use of categories or examples provided to the system in the form of labeled data (e.g., images). In this way, the problem becomes that of learning a representation or mapping that is able to generalize to previously unseen data. Early examples of these are the prediction of *hidden* affordances such as containment or sittable based on multiple classifiers and CAD object models in Aldoma et al. (2012), learning a logistic regression *pushable, liftable* and *graspable* affordances to improve object segmentation in Kim and Sukhatme (2014), learning object functional classes in Hinkle and Olson (2013) or *containability* in Yu et al. (2015) based on physics simulations. Tool-part or object-part affordances have also been studied using this approaches based on collections of labeled data; for instance Myers et al. (2015) and Rezapour Lakani et al. (2018) associated local shape and spatial information to affordances such as grasp, scoop, support, pound, and cut. In Abelha and Guerin (2017), tool-part affordances are used to find substitute tools to accomplish a specified task by matching against a database of manually-defined object-task pairs.

More recently, a group of these approaches take advantage of large collections of labeled data using deep learning methods. These approaches treat affordance perception and learning as a segmentation problem in computer vision and typically leverage the ability of Convolutional Neural Networks (CNNs) to learn features from annotated data in a fully-supervised manner (Nguyen et al., 2016; Porzi et al., 2017) or a weakly-supervised approach (Sawatzky et al., 2017). Further development of such affordance segmentation works has included object detection (Nguyen et al., 2017) with larger datasets of RGB images, as well as object detection and recognition using multi-stream deep neural networks (Do et al., 2018) to increase the affordance detection performance. Deep CNNs were also used for scene functional-region prediction by Ye et al. (2017), leveraging object detection and recognition architectures first to generate thousands of region proposals that are then classified according to functional types such as *sittable, turn on/off* , and various types of grasp.

### 2.2. A Critical Review of Affordance Research

Although there have been many interpretations and debate regarding the true nature of the affordance concept, to a certain extent, roboticists seem to agree that affordances should be a relation between two (or more) interacting entities. Many approaches learn these relations as a symbolic representation that enable a robotic system to plan its actions; while many of these approaches allow for human-inspired learning stages, the applications are limited to a small set of objects and affordances. It is not clear how the models would apply for novel objects and novel realistic environments.

A similar dilemma is faced by methods that solely focus on one type of interaction, i.e., *grasping* or *human* affordances. In spite of the remarkable progress in categorization-free affordance learning (see Zech et al., 2017 for a recent comprehensive review) the question remains open about the generalization of the approaches for other types of interaction or scenarios that do not require manipulation.

Notably, within the last couple of years, a notorious trend has been observed for learning object or tool affordances, that is the exploitation of large collections of labeled imagery. One important challenge faced by these methods is that they usually need to detect (and even recognize) object instances in the environment; whereas deep neural networks have proved to be a powerful tool in this area, generalization and scalability remain as important challenges. For instance, the models would need new annotated data and an extensive retraining process in order to learn new affordances. Besides, the manually annotated datasets used for training build on the assumption that objects in the environment have a pre-defined set of affordances; making uncertain how an agent would discover new affordances, i.e., the absence of annotated data.

In terms of perception, many earlier approaches tackled the problem by fitting hand-crafted features (e.g., color, shape, size) to encode and identify objects properties and placement (Stoytchev, 2005; Montesano et al., 2007, 2008; Moldovan et al., 2012; Moldovan and De Raedt, 2014b). Other methods have leveraged local 3D shape or surface features computed over pointclouds to represent objects (or object parts) in the environment; for instance (Aldoma et al., 2012; Kroemer et al., 2012; Detry et al., 2013; Kaiser et al., 2014; Kim and Sukhatme, 2014; Pas and Platt, 2016). As stated previously, more recently, perception systems have used machine learning methods over large amounts of data to learn features *directly* from the input data (Nguyen et al., 2017; Do et al., 2018) to identify *classes* of objects. Overall, works investigating affordances have used a variety of features computed usually from visual information. Remarkably, the representation that remains present across most approaches is the shape or geometrical information. We believe that the perception of affordances based on 3D geometrical information is a far more promising generalization alternative than attempting to semantically categorize entities in the world; after all, geometry of everyday objects is what strongly dictates the physical interactions that are possible with the environment.

### 2.3. Context of Our Contributions

In this paper we show that methods based entirely on geometric information are capable of predicting high quality and meaningful affordance locations for realistic environments. In contrast to other works considering geometric information for affordance perception (Kaiser et al., 2014, 2015, 2016, 2018; Pas and Platt, 2016, 2018), the approach that we propose does not rely on higher-level geometric primitives nor complex features computed on the environment (e.g., planes, cylinders, cubes, etc.). Moreover, the general purpose nature of the representation that we propose allows to characterize affordances for *simple* objects such as a placing a mug but also enables the representation of more complex interactions like a human riding a motorcycle. Here, we extend our previous work Ruiz and Mayol-Cuevas (2018) to allow several dozens of interactions to be characterized with an unified and scalable affordance representation.

Contrary to methods in Computer Graphics that study functionality and shape (Zhao et al., 2014, 2016, 2017; Hu et al., 2015), our approach takes into account visually perceived information. We adopt a pointcloud representation which allows us to work with data generated from robotics sensors such as RGB-D cameras, avoiding the need for fine-grained geometries and detailed mesh information that are typically exploited in Computer Graphics. Furthermore, by introducing the concepts of affordance keypoints and provenance vectors, we devise a representation that is straightforward to compute and tolerates well changes in geometry. This provides good generalization to unseen scenes from a single example and enables to perform affordance detection at high framerates, a key requirement for robotic systems.

Inspired by recent deep learning architectures able to operate in the 3D domain (Qi et al., 2017b), here we address key limitations of the above approaches by incorporating a deep 3D saliency detection mechanism to reduce spatial search and computation needed to produce affordance predictions. This adopts a data-driven approach, yet without forcing affordance categories as such. This enables us to significantly speed up the selection of potential affordance candidate regions compared to our previous work.

Finally, we demonstrate that we can significantly outperform the baselines in terms of performance and speed, and we validate our affordance predictions with human judgement.

## 3. Our Approach to Geometric Affordance Determination

There are three main components of our approach: (i) the computation of the geometrical representation of the affordance between a pair of objects (section 3.1), (ii) the agglomeration of these representations for multi-affordance estimation (section 3.2), and (iii) the incorporation of deep saliency for fast scene parsing (section 4).

Our approach starts by computing what we call the Interaction Tensor for an interaction that takes place between any two objects. The approach is based on the Bisector Surface (Peternell, 2000); but more specifically, we take inspiration from the concept of Interaction Bisector Surface (IBS) that has been successfully exploited within the Computer Graphics community (Zhao et al., 2014). In short, the Bisector Surface for any two geometric objects is defined as the locus of equidistant points between the objects. The IBS is a generalization of that locus when it is computed between two or more 3D models in a scene. Importantly, the IBS can be approximated by computing the Voronoi diagram between objects. In this sense, the IBS is the set of points equidistant from two sets of 3D points (i.e., two objects). In this section, we summarize the core aspects of our affordance descriptor; then we introduce the method that allows us to represent multiple affordances and the algorithm to perform one-shot prediction.

### 3.1. The Interaction Tensor

**Figure 2:**
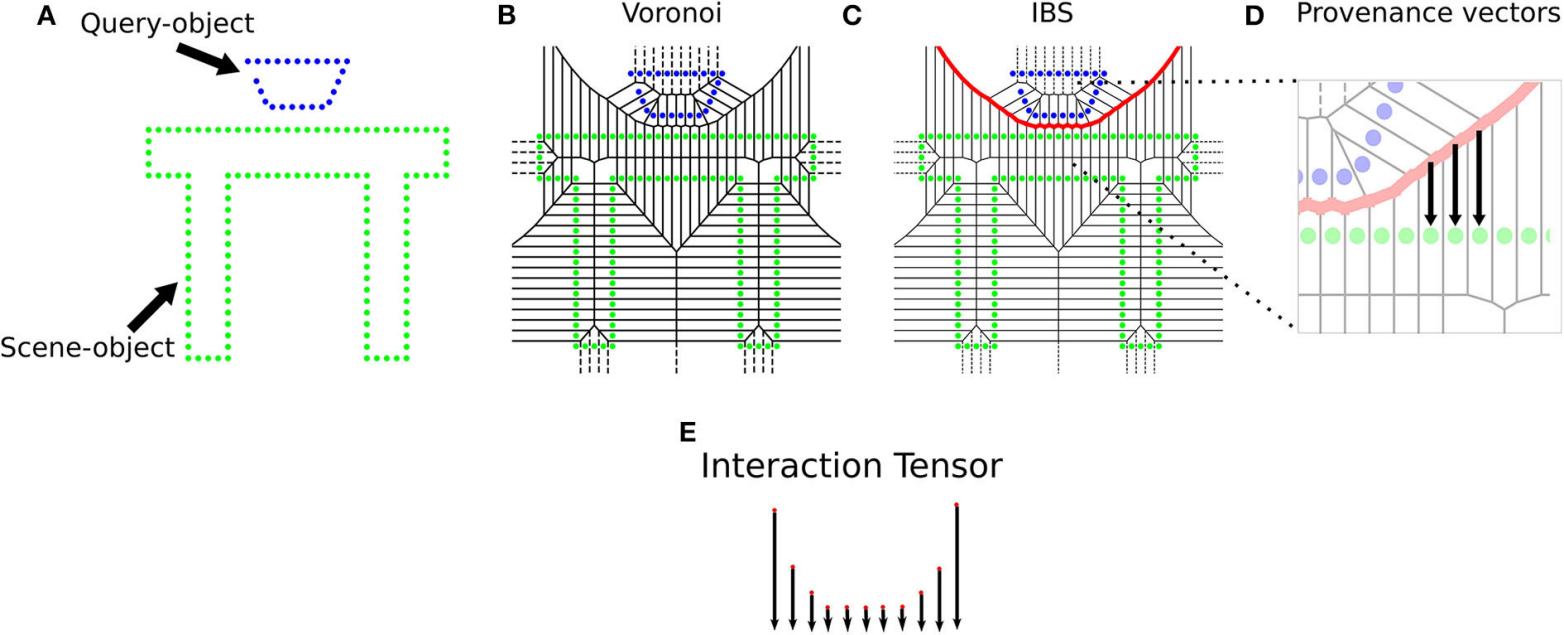
Computation of an Interaction Tensor for *Placing* a bowl. **(A)** Simplified 2D illustration of the interaction, **(B)** Voronoi diagram over all points, **(C)** IBS shared between interacting objects, **(D)** Computation of provenance vectors, which go from points on the IBS to its closest Voronoi cell centroid in the scene-object. **(E)** Interaction Tensor formed by affordance keypoints (3D points and vectors).

**Figure 3:**
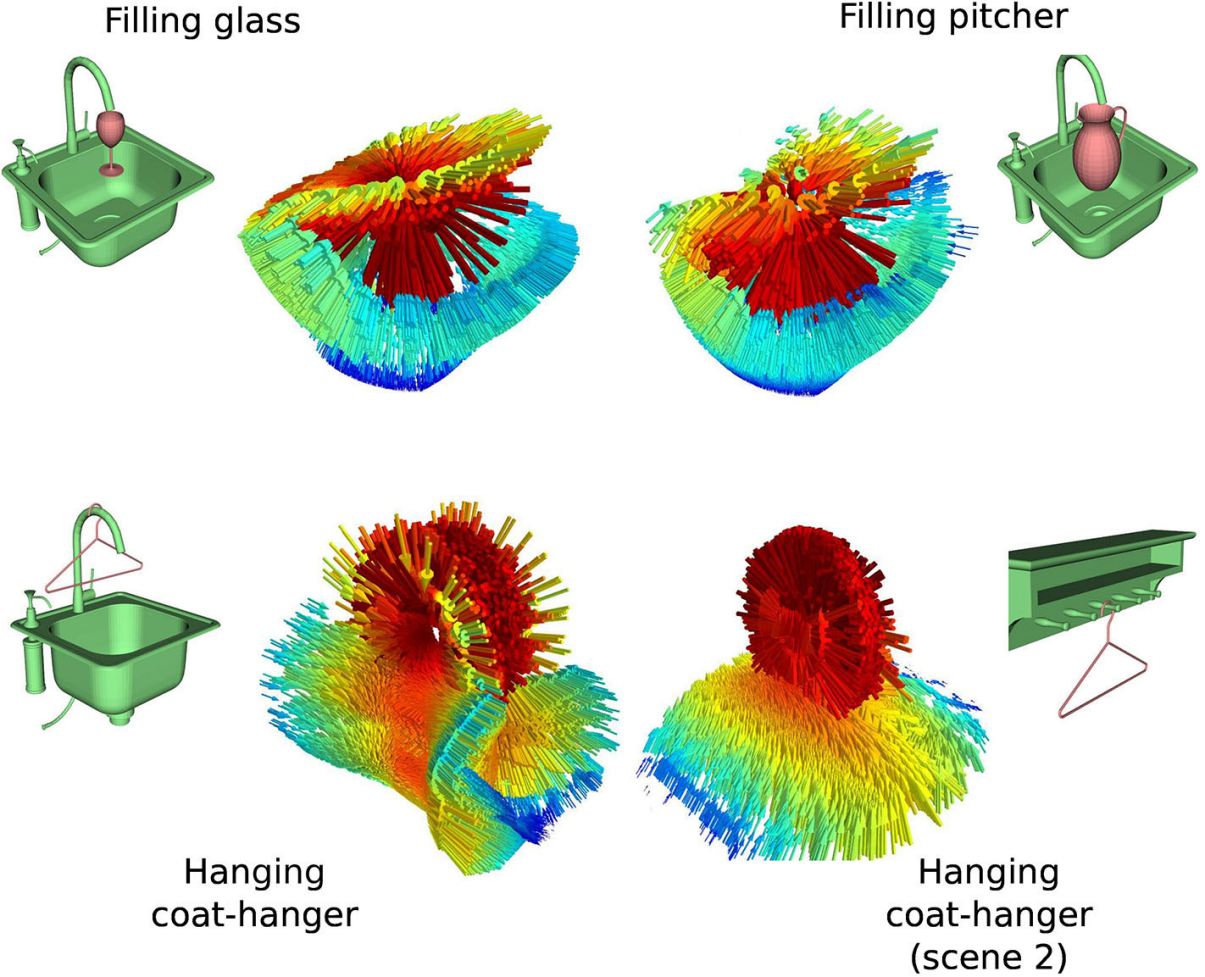
iT examples for a subset of the objects in our study. Top row shows iT examples for *Filling* objects with different geometries. Bottom row shows iT examples for *Hanging* the same object (coat-hanger) on different scenes (i.e., scene-object). Despite the changes in the geometries involved in the interaction, the tensor shows similar features across affordances. Colors in the tensors represent weighting scheme (red is higher). Arrows in vector fields are re-scaled to more clearly display similarities.

The Interaction Tensor (iT) is a vector field representation that characterizes affordances between two arbitrary objects. The key steps include an example affordance from e.g., a simulated interaction, the computation of the IBS between an object (query-object) and scene (or scene-object) using dense pointclouds (from CAD models), and estimating provenance vectors. These vectors are used in the computation of points on the bisector surface and they go from points in the IBS to their closest neighbor in the scene. The top row in Figure 2 illustrates the elements and the process involved in the computation of an affordance iT for any two given objects. For practicality, every iT is expressed in scene-object frame coordinates, i.e., we set the origin of every iT's reference frame to the point in the scene-object that is closest to the query-object during *training*. For instance, the descriptor for *Filling*-glass in Figure 3 has its frame origin in the faucet spout. As another example, the *Placing*-bowl descriptor of Figure 2 would have a reference frame with origin on top of table where the bowl rests.

A descriptor for any given affordance is obtained by sampling *N* *affordance keypoints* (3D point and provenance vector) from the iT example, each one of these keypoints has a weight that encodes the relevance of that particular location for the interaction. The representation is fast to compute and compact since only the affordance keypoints and provenance vectors are used to describe the interaction, neither query-object, scene-object nor bisector surface are needed afterwards. Examples of iT descriptors for the type of affordances that we study are shown in Figure 3, where weights are depicted as the colors in the vector fields (red is higher). The weighting scheme follows the intuition that regions of the interaction where objects come close together should be more important (higher weight) for the interaction, as opposed to those regions where the interacting objects are further apart. As in our previous work Ruiz and Mayol-Cuevas (2018), we leverage the weighting scheme to sample the keypoints that form a descriptor. A keypoint *x*_*i*_ is sampled from the dense iT with probability proportional to its associated weight, $P\left(x_{i}|w_{1},w_{2},\ldots ,w_{n}\right)=\frac{w_{i}}{\overset{n}{\underset{d=1}{\sum }}w_{d}}$. This sampling method follows the same intuition that locations where objects come closer together or touch are highly relevant for the interaction, thus resulting in higher keypoint density in those regions.

Figure 3 also serves to illustrate the robustness of the iT representation to changes in the geometry of the interacting objects, the top row in this figure illustrates an interaction (*filling*) with two different query-objects, whereas the bottom row in the same figure depicts descriptors for another interaction (*hanging*) with two different scene-objects. Note how the overall geometry of the descriptor remains similar across interactions (i.e., horizontally).

### 3.2. Interaction Tensor Agglomeration

Our approach for scalable multiple-affordance detection, which we call iT agglomeration, follows a one-shot learning approach, i.e., it uses a single example from every affordance to devise the multiple-affordance descriptor. With such representation and algorithm, one can give an answer to questions such as “*What can I afford to do here?*” on multiple point locations of an input scene without the need to individually test affordances. The approach allows to increase the number of affordance-object pairs queried simultaneously at test time without heavily compromising detection rates.

**Figure 4:**
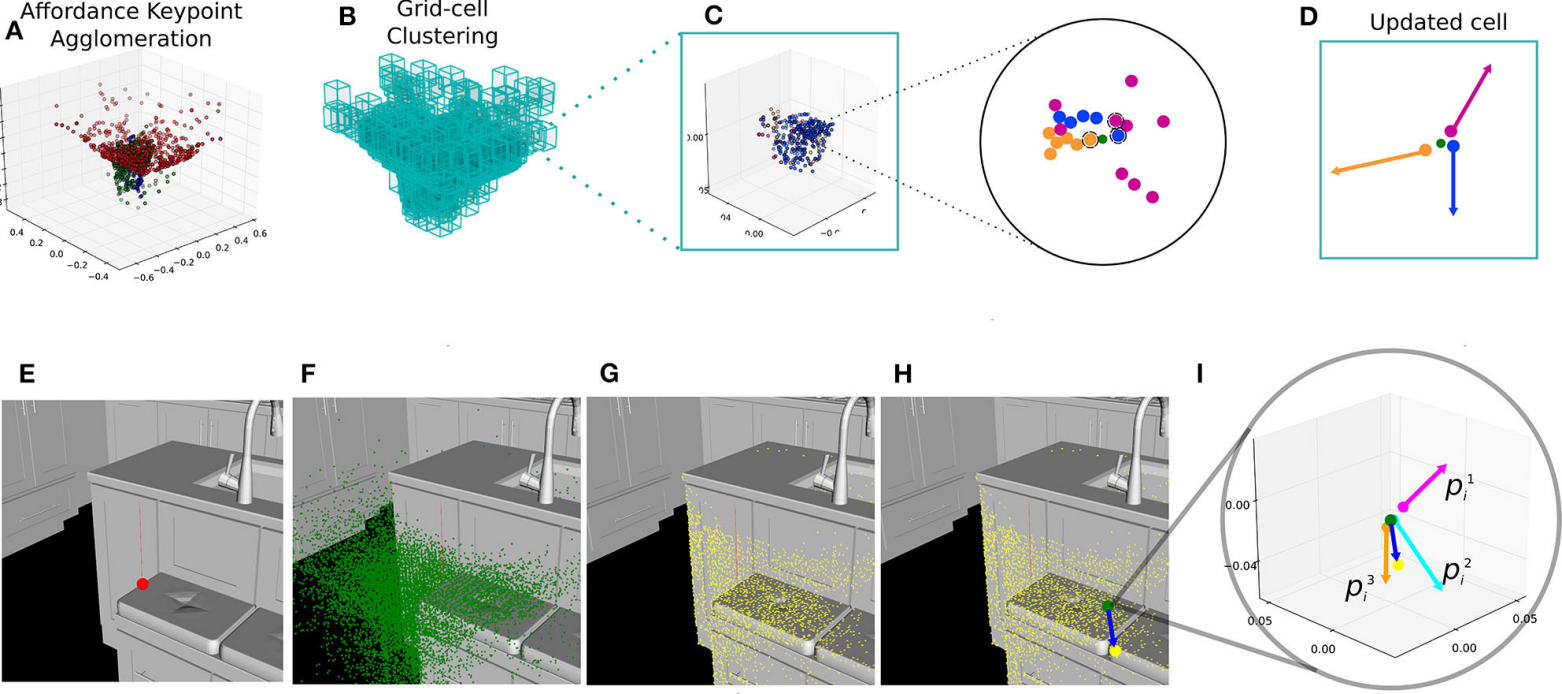
Top row illustrates the computation of an agglomerative descriptor for 3 affordances. **(A)** Single-affordance keypoints are agglomerated (affordances shown as different colors), **(B)** uniform-size cell grid is fitted to the pointcloud, **(C)** one cell can potentially contain many keypoints from multiple affordances, only the closest keypoint (per-affordance) to the cell centroid (green) is taken into account during the update process, **(D)** an updated cell with the provenance vectors associated to the keypoints kept after clustering. Bottom row sequence illustrates our affordance prediction algorithm. **(E)** A test-point is sampled from the input scene (red), **(F)** The agglomerative representation (green) is aligned relative to this test-point, **(G)** The 1-NN in the scene (yellow) for every centroid in the agglomeration, **(H)** An example test-vector (blue) from a cell centroid to its closest scene point, **(I)** A test-vector is compared against the stored provenance vectors $p_{i}^{k}$ associated with affordance keypoints in that cell. In this particular cell, 3 scores are obtained.

As the name suggests, our method for multiple affordance representation accumulates or agglomerates several individual affordance descriptors in a single pointcloud. We start by computing as many single-affordance descriptors as required, i.e., one for every interaction of interest. Once all descriptors have been computed with the iT, we agglomerate them in a single pointcloud. Note that all the descriptors share the same coordinate frame, which is relative to a point in the scene-object where the simulated interaction took place (i.e., training as explained in section 3.1). Therefore, all the trained descriptors are readily aligned. Agglomerating these descriptors is as straight-forward as aggregating them into a single bigger pointcloud. Once this single bigger pointcloud has been obtained, we perform clustering by first fitting a grid of uniform-size cells covering every single *affordance keypoint*. Then, we use as seed-points only the centroids of non-empty cells. For every one of these cells, we only keep the keypoints that are closest to the centroid in a per-affordance basis. For instance, one cell could contain 100 keypoints, all coming from the descriptor of *Placing-bowl*; after the iT clustering process is carried out, this cell will only contain the keypoint that is closest to the cell's centroid. Finally, we update the centroid location using the keypoints within each cell, keeping track of the *provenance vectors* associated with them as well as the number of keypoints from each affordance in each cell. We attempted more sophisticated ways to learn the agglomeration but found the above straight forward method to be better and faster. The top row in Figure 4 depicts the cell-updating process for iT clustering algorithm. The clustering process leads to a reduced number of 3D points (cell centroids) that represent a large number of affordance keypoints. This reduced number of new keypoints and their associated *provenance vectors* are used to compute and predict affordance candidate locations at test time.

### 3.3. One-Shot Prediction

Previous methods employing the IBS for functionality analysis in Zhao et al. (2014), Hu et al. (2015), Zhao et al. (2016), Pirk et al. (2017) have relied on the computation of local shape features on the bisector surface, these features are then fed to a machine learning algorithm (or similarity function) in order to retrieve or synthesize similar interactions. Then, for any new pair of objects, they need first to perform many costly computations to make a prediction; namely, estimate the IBS with dense pointclouds and remove noisy data, compute shape and topological features at various locations using mesh information, and (typically) build histograms representing the global shape features. Our method differentiates from these previous works by instead approximating the **iT** descriptor at prediction time (i.e., *testing*) via a Nearest Neighbor (NN) search; and then *directly* comparing against the descriptor from the *training* example. This comparison can efficiently investigate whether there exist relevant regions such that the same (or a similar) iT can be computed in a testing location. Notably, our prediction algorithm requires a single *training* iteration per affordance. Moreover, our method does not make use of object data (e.g., query-object), it only requires the descriptor to predict an interaction in a new scene.

First, in order to estimate the likelihood of an affordance for a new test point in an input unknown scene the descriptor needs to be aligned relative to that new location. This is achieved by simply applying a translation; multiple orientations can be also tested by applying the corresponding rotation. These operations are straightforward to compute due to the fact that the descriptor is already expressed *w.r.t* a point in the scene; therefore, the pose of the affordance descriptor *X*_*a*_ given a test-point *t*_*i*_ is given by

(1) $$\begin{array}{l} X_{a}^{\prime }=R_{o}T_{t_{i}}X_{\text{a}} \end{array}$$

where

$$\begin{array}{l} R_{o}=\left[\begin{array}{cccc} \text{cos }\theta & \text{sin }\theta & 0 & 0\\ -\text{sin }\theta & \text{cos }\theta & 0 & 0\\ 0 & 0 & 1 & 0\\ 0 & 0 & 0 & 1 \end{array}\right],T_{t_{i}}=\left[\begin{array}{cccc} 1 & 0 & 0 & t_{i_{x}}\\ 0 & 1 & 0 & t_{i_{y}}\\ 0 & 0 & 1 & t_{i_{z}}\\ 0 & 0 & 0 & 1 \end{array}\right] \end{array}$$

where θ is the angle at which the interaction should be tested and 〈*t*_*i*_*x*__, *t*_*i*_*y*__, *t*_*i*_*z*__〉 are simply the *x, y, z* coordinates of the testing point in the scene. This operation allows us to predict affordance candidate locations at different several orientations instead of only the one used for the *training* example.

Once the descriptor is aligned relative to the test point, the NN-search is performed to estimate test-vectors. This is done by computing the 1-NN in the scene for every *keypoint* in the descriptor. These vectors are the approximation of *provenance vectors* at test time and they are compared in order to estimate the likelihood of the interaction via Equation (2) shown below:

(2) $$\begin{array}{l} s^{k}=\frac{1}{N^{k}}\overset{N^{k}}{\underset{i=1}{\sum^{\;}}}\frac{1}{\sqrt{2\pi {\left(w_{i}^{k}\right)}^{2}}}e^{-\frac{{\left(\Delta_{i}^{k}\right)}^{2}}{2{\left(w_{i}^{k}\right)}^{2}}}, \end{array}$$

where

$$\begin{array}{l} \Delta_{i}^{k}=\frac{∥{\vec{v}}_{i}-{\vec{p}}_{i}^{k}∥}{∥{\vec{p}}_{i}^{k}∥},\;w_{i}^{k}=1-\frac{\left|{\vec{p}}_{i}^{k}\right|}{\left|{\vec{p}}_{max}^{k}\right|} \end{array}$$

where *N*^*k*^ is the number of keypoints in affordance *k*, $w_{i}^{k}$ is the weight of the i-th keypoint of affordance *k*, computed proportionally to the magnitude of its corresponding *provenance vector* ${\vec{p}}_{i}^{k}$ (*i*−th keypoint of affordance *k*). $\Delta_{i}^{k}$ is the difference between vectors ${\vec{v}}_{i}$ and ${\vec{p}}_{i}^{k}$, where ${\vec{v}}_{i}$ is the test-vector estimated with the centroid of the cell containing the i-th keypoint. Equation (2) uses a Gaussian function to measure the difference between vectors (magnitude and orientation), where the acceptable variance is inversely proportional to the keypoint's weight $w_{i}^{k}$. The inclusion of weights in the function allows to relax the matching criteria for regions of the interaction that are not very relevant (low weight), and stricter criteria for those regions that are more relevant (i.e., higher weight). Note that for single-affordance predictions (section 3.1) the approach remains the same, since for that case *k* = 1.

The bottom row in Figure 4 illustrates the steps followed to make affordance predictions with our proposed approach. Following these steps allows to obtain a score (likelihood) for every affordance for any given location in a scene; we can then establish the threshold that produces the optimal predictions. We do so by asking humans (via crowdsourcing) to evaluate the predictions made by our algorithm at different scores. The specifics on the method to obtain the optimal score for prediction can be found in Ruiz and Mayol-Cuevas (2018) and Ruiz and Mayol-Cuevas (2019). In short, we chose the value that achieves the best accuracy on human-evaluated predictions of multiple affordances.

Notice that the search performed in the scene, i.e., the way in which test-points are selected, involves sampling from the input scene uniformly trying to test locations across the whole input. It should be kept in mind that no prior assumption is made regarding features on the scene, objects appearance nor complex surface features *likely* to afford a target interaction. In other words, the approach that we propose is agnostic to shape and appearance features computed in advance and instead estimates the likelihood of the affordance by *hallucinating* the interaction at test-time. Also note that our prediction algorithm does not make use of the query-objects. Our one-shot affordance prediction only requires an input scene (to search in) and the multiple-affordance descriptor.

Naively, one could detect several affordances by testing individual descriptors one after the other. However, this quickly becomes a problem; for instance, in order to detect k affordances (with N keypoints each) at Θ orientations in a single location, one would need to search for *k* × *N* × Θ nearest neighbors. Effectively what we propose is to take advantage of the overlapping pattern found when many affordance descriptors are agglomerated in a single location, which leads to a great reduction in the dimensionality of the representation and a speed-up of nearly six times over the previous method (Ruiz and Mayol-Cuevas, 2019).

## 4. Learning Affordance Saliency

The Interaction Tensor is a dense representation that characterizes the interaction between pairs of objects. Our approach for one-shot affordance prediction relies on *keypoints* that are sampled from the tensor to form a descriptor. As described earlier, we base the sampling of keypoints on the intuition that the 3D regions of the interaction where objects are close to each other are more important for characterizing the interaction, i.e., weight-driven sampling. In previous work, and as demonstrated later in section 5, we show that a sparse and empirically-found sampling size works well. In this section we present our investigation regarding the use of a state-of-the-art deep learning method to optimize a multiple-affordance representation for 3D data, i.e., pointclouds. In short, we capitalize on the PointNet++ architecture (Qi et al., 2017b), which has shown noticeable results for 3D shape classification tasks. This architecture is advantageous in our problem mainly for two reasons: (1) its ability to capture local structures induced by the metric space 3D points live in, and (2) its robustness against non-uniform sampled pointclouds. In short, instead of naive scene sampling, we employ the network's abstraction power and learn the specific locations in the input (scene) that are used for interaction prediction.

### 4.1. Deep-Learning Affordance Pointclouds

Ideally, we want to learn an affordance representation that efficiently accounts for the presence of multiple interactions in a given scene. In our work, scenes are represented with pointclouds, sensed with RGB-D cameras or computed in simulations from a CAD model. Therefore, we want to be able to predict all the possible interactions that any given pointcloud affords, i.e., all the affordance *classes* in a pointcloud. This contrasts with traditional approaches for deep learning on 3D shapes, where the studied pointclouds belong to a single class or type. In our investigation, a single pointcloud can belong to multiple “classes” or affordance categories at the same time. In order to learn affordances in such a scenario, we formulate the learning problem as a multi-label multi-class classification; to this end, we modify the PointNet++ architecture by replacing the last softmax layer with a sigmoid layer. This adaptation to the architecture allows us to obtain independent scores or probabilities for every interaction that a pointcloud might afford.

A second modification to the network architecture includes changing the sampling regions that are used to learn features at different hierarchies and pointcloud densities. Briefly speaking, there are three main layer types in the original PointNet++ architecture: Sampling, Grouping, and PointNet. Sampling layers select points (from the input) that are used as centroids of the regions where features should be extracted. Grouping layers gather additional data by sampling points within local regions (computed by the previous layer), these local regions and their centroid data are concatenated to build new (internal) point sets which form a hierarchy. Finally, PointNet layers is where the data from every region is encoded in the form of feature vectors by a series of convolutions and pooling operations. In the original implementation, the size of the local regions in Grouping layers is set by a fixed-radius sphere centered at each local region. We modify the network to allow the sampling regions to change on-line in proportion to the input size instead of being of a fixed radius. Specifically, we allow the value of these parameters to change in proportion to 20% (at the lowest-level layer) and up to and 40% (at the highest) of the bounding box of the pointcloud at the input.

This follows from the fact that, as in most pointcloud classification approaches, PointNet++ normalizes the input data to a unit-sphere or unit-box, where having sampling regions with fixed sizes is acceptable. However, this is not feasible in our learning approach given that we work with real-world scales. For instance, having a pointcloud of a chair of 1 meter-height is substantially different from a *toy chair* with a height of 10 cm; the latter would not afford *Sitting* for a human. For this reason, we modify the architecture to allow the sampling regions to change proportionally to the current training pointcloud.

We refer to this modified version of the deep network as m-Pointnet in the remainder of this paper. Specific details on how we train such network are described in the Results and Evaluation section (section 5).

### 4.2. Scene Saliency From Affordance Predictions

Once the network has learned to detect multiple affordances in a given pointcloud we leverage its data abstraction capabilities by finding the regions in the input that the network uses to make a prediction. This is achieved by keeping track of the points that activate neurons the most. These points suggest salient 3D locations in a given pointcloud and are analogous to the concept of critical pointsets presented by Qi et al. (2017a). More specifically, once the network has learned to predict correctly multiple affordances per pointcloud, we let the network go through the data again and we keep record of the 3D locations at the input that give the maximum value in the lowest-level feature map, i.e., the max-pooled features at the lowest-level PointNet layer of the network. The network utilizes the features pooled from those locations as a base for higher-level features and, in turn, to compute the feature vector used to correctly classify the affordances in a given pointcloud. We refer to these salient regions in the input scene as scene saliency in the remainder of this paper. Recall that we zero-mean our pointcloud dataset; therefore, we can readily accumulate (via voting) the salient 3d regions across multiple pointclouds. We choose as final scene saliency the 3D locations that activated (accumulated votes) at least 50% of the time.

**Figure 5:**
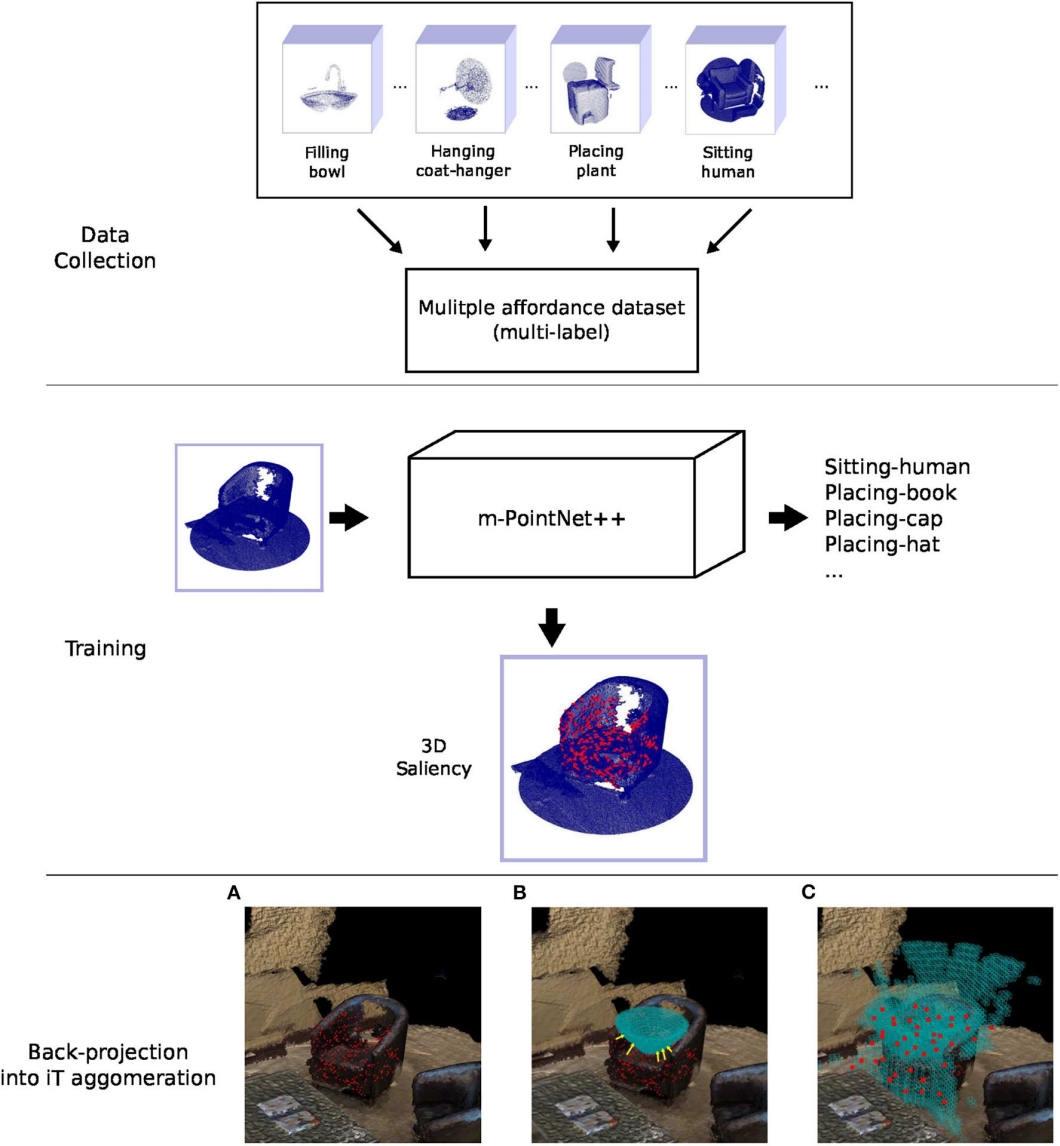
**(Top)** The modified version of PointNet++ is trained with multiple affordance labels per training example. This dataset is built using the single affordance predictions of the iT method. **(Middle)** We use the salient regions learned by the network (shown in red) in order to learn to sample from the interaction tensor. These regions are used by the network during training/validation to correctly classify pointcloud instances. **(Bottom)** shows the back-projection of saliency into iT agglomeration. **(A)** Scene saliency (red points) for an example pointcloud. **(B)** The nearest neighbor in the agglomeration (pointed by yellow arrows) is computed for every salient point (red points) in the scene. **(C)** This projection is carried out for every pointcloud in the dataset, the new affordance keypoints (red points) are comprised of the cell centroids that received projections the most.

Due to the fact that the iT relates points in the two interacting objects (i.e., scene and query-object), we can simply project salient points (from the scene) learned by the network back into their associated iT location. Briefly speaking, for all scene-salient points, we compute the nearest-neighbor in the iTs of all the interactions afforded by the current pointcloud. This is the inverse process to the one shown in Figure 2D. Given that the iTs are very dense entities we use a grid representation (i.e., cell grid) to alleviate the back-projection process (blue cells in Figure 5: Multiple-affordance representation). Once all salient locations have been projected into their associated iT, we create a new multiple-affordance descriptor by considering the locations in the iT agglomeration (i.e., cells) that *received* projections the most. Figure 5 illustrates the general idea behind our approach to learning an affordance representation from scene saliency.

## 5. Experiments and Results

Our investigation focuses on the perception of a subset of affordances for indoor environments and on the algorithms that give robots the ability to answer the perceptual question of “What can I do here?” or “What can I afford to do here?.” The general idea of our experiments is to apply our one-shot prediction algorithm on an input 3D scene (pointcloud) as introduce earlier in section 3.3 (bottom row in Figure 4). These experiments consider 84 interactions, which include multiple household objects from a wide range of geometries and dimensions such as mugs, cutlery, hammers, etc, and is inspired by standard robotic manipulation datasets such as Calli et al. (2015). We also include human 3D models to investigate *human* affordances such as Sitting. The descriptors for all these interactions are computed as detailed earlier (section 3.1) with pointclouds from CAD models and simulating the target interactions. Similar to previous work (Ruiz and Mayol-Cuevas, 2018), the dense pointclouds are generated by sampling points on every face (i.e., triangle) of the CAD model with $d=\left(1-\sqrt{r_{1}}\right)\text{a}+\sqrt{r_{1}}\left(1-r_{2}\right)\text{b}+\sqrt{r_{1}}r_{2}\text{c}$, where *d* is a point sampled on the triangle defined by vertices *A, B* and *C* (with coordinates *a, b, c* respectively) and where *r*_1_ and *r*_2_ are random numbers sampled from a uniform distribution between [0, 1]. The randomness allows to compute more realistic pointclouds where point distribution is not uniform, as opposed to alternative methods such as e.g., Poisson disk.

All the affordances that we study are of the form *Place-book, Hang-umbrella, Sit-human*, etc. Keep in mind that some objects afford more than one interaction e.g., *Fill-Pitcher* and *Hang-Pitcher*. Also note that it is possible to consider some top-level clustering with conventional generic labels for affordances such as *Placing, Hanging, Filling*. The complete list of CAD models for our object-affordance pairs can be found along the x-axis in **Figure 8**.

It is worth reminding that our algorithm does not need the *training* objects (query-object nor the scene-object) to make a prediction. Prediction is made using a new input scene and only the descriptor of the interactions, this descriptor suffices for the NN-search approximation. The qualitative results that we present in this section show the query-objects (shown in green) only to visualize more clearly the predicted interaction.

### 5.1. Saliency Training and Data Collection

For these experiments we use the descriptor learned via 3D scene saliency; thus we first train the saliency network (m-PointNet++) with the scene dataset presented in Ruiz and Mayol-Cuevas (2018), that comprises pointclouds of indoor environments from synthetic data and real RGB-D scans. In order to build a dataset suitable for training the network, we first run the iT prediction algorithm for all the affordance-object pairs individually. By default, the output of the algorithm is the likelihood (score) of a point location in the scene of affording the interaction. The pointcloud that actually allows the interaction to take place in that location is obtained by extracting the voxel surrounding the test point detected as a good location. With this procedure, we form a dataset of 10K pointclouds per interaction. Additionally, we generate background or “negative” examples from affordance detections of scores lower than the optimal value (i.e., *s*_*k*_ = 0.5). We then process these predictions to detect pointclouds that share multiple affordances and automatically produce the corresponding annotations for multi-class multi-label classification.

The 3D affordance dataset is comprised of 918K pointclouds (10K per-affordance on average) with an 80/20 split for training and validation. Data augmentation is performed on-line by rotating the pointclouds around the vertical axis, adding jitter and randomizing the points sampled at the input. Training is carried out with cross-entropy $L=-\overset{k}{\underset{i=1}{\sum }}y_{i}log\left(\hat{p_{i}}\right)+\left(1-y_{i}\right)log\left(1-\hat{p_{i}}\right)$ with *k* = 85 (84 affordances and background). Additionally, we perform *L*_2_-norm regularization since over-fitting was observed during preliminary experiments. In order to train the network, we zero-mean the pointclouds, which allows us to track scene saliency for different pointclouds relative to the same reference frame.

### 5.2. Implementation Details

Training for the m-PointNet network is carried out using the Adam optimizer with initial learning rate of 0.001, momentum 0.9, batch size 32, and a decay rate of 0.7. Batch normalization is used for every fully connected layer and dropout with a keep ratio of 0.5. Training is carried out for at least 250 epochs and until convergence is achieved.

For prediction, we leverage GPU parallelization to predict multiple affordances at multiple orientations in a single iteration of our prediction algorithm. We achieve this by using a bigger descriptor that includes multiple orientations; in other words, by concatenating the affordance descriptor of Θ = 8 orientations [evenly distributed in [0, 2π)] into one bigger descriptor. This implementation allows us to compute the score for 8 orientations of all affordances in a single iteration of the prediction algorithm, e.g., a GPU core computes the score for affordance *k* = 1 at θ = 0^*o*^, another for affordance *k* = 2 at θ = 90^*o*^, etc.

Training and prediction are performed on a desktop PC with a single Titan X GPU.

### 5.3. Evaluations

Our evaluations include the effect that the parameters of our algorithms have over the prediction rates and performance. We present results of our baseline algorithm, i.e., iT Agglomeration (as described in section 3.1) and our method that leverages saliency to improve the agglomerative approach, denoted as “iT + Saliency” for short. We also evaluated the performance of the adapted PointNet++ architecture when tested on its own, i.e., as in a *standard* shape classification task. These results are shown as “m-PointNet++” throughout the following subsections.

**Figure 6:**
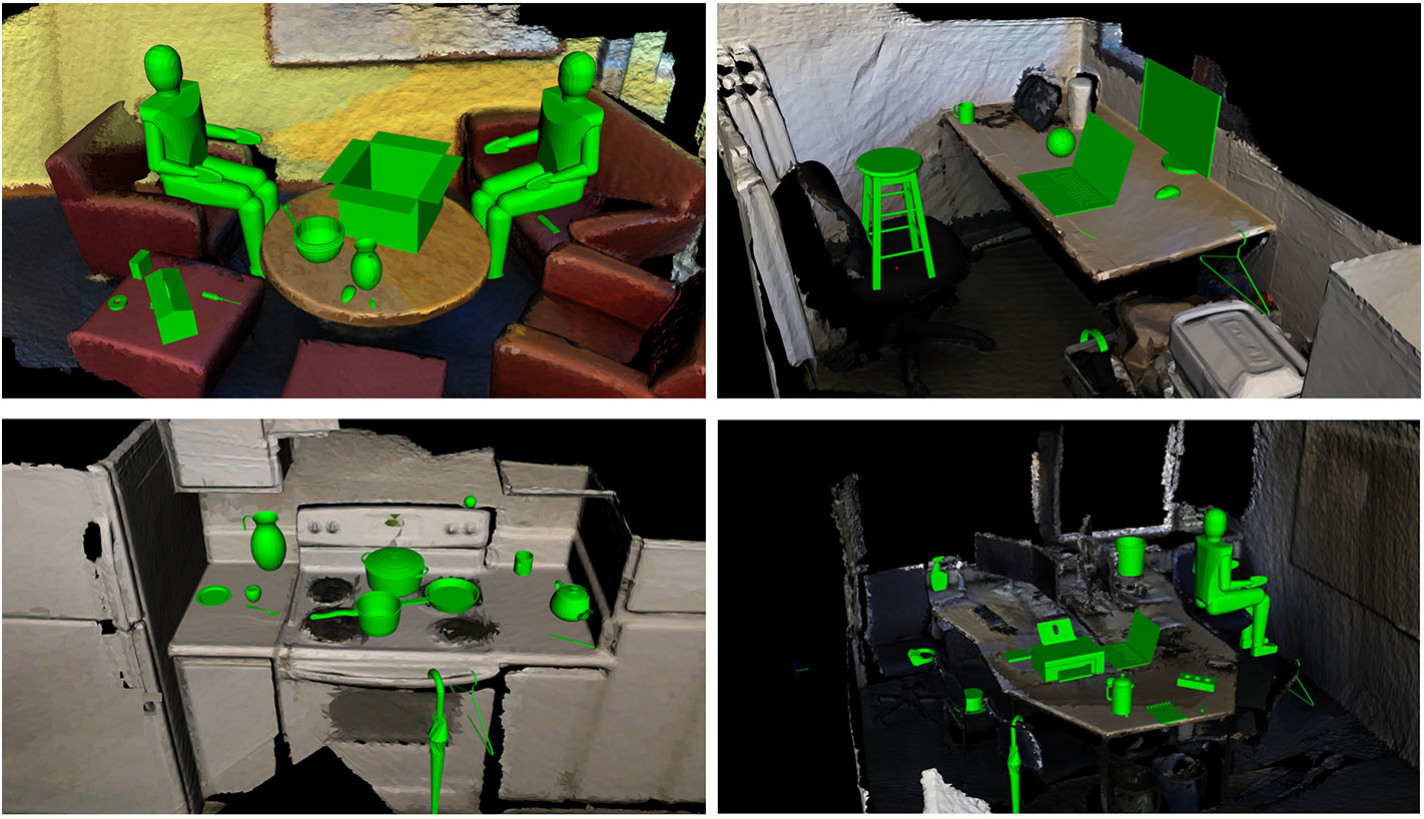
Multiple-affordance detection on never before seen RGB-D environments made with our saliency-based method. Note those locations of not achievable actions such as the coat hanger going through the oven's window are due to the inaccuracies and challenges from the RGB-D sensor data. Most other detected affordances are achievable despite the fact that they are obtained without a script or any other prior scene information.

We test and show examples of our predictions on 150 scenes (randomly selected) of ScanNet (Dai et al., 2017), comprising living rooms, kitchens, dining rooms and offices. Qualitative results of our predictions are shown in Figures 1, 6, 8 throughout the paper. In these tests, we predict up to 84 affordances or interaction possibilities in any given location of an input scene.

#### 5.3.1. Optimal Detections

**Figure 7:**
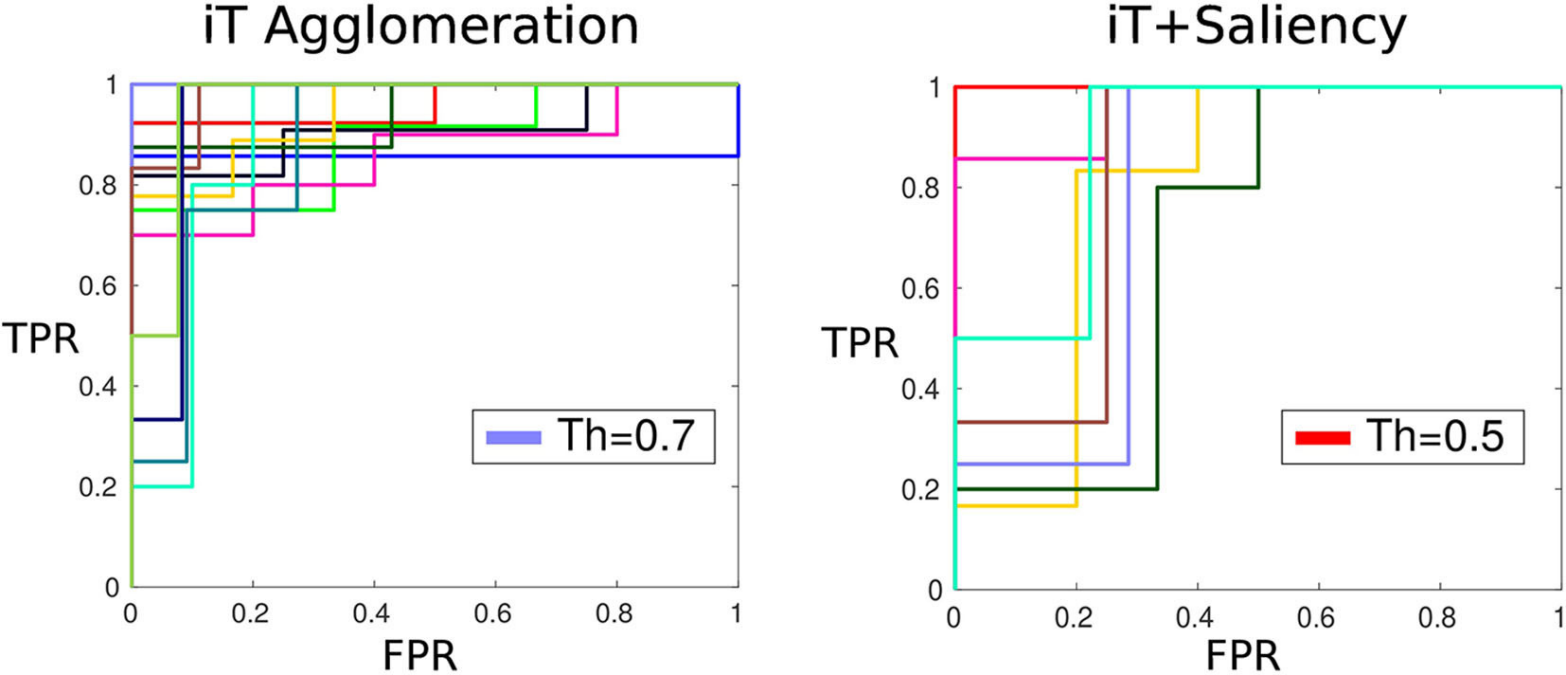
Mechanical turk evaluation. ROC plots (True Positive Rate TPR vs. False Positive Rate FPR) show the family of classifiers generated by setting different thresholds bands to the prediction score. Both approaches show a similar performance: the affordance predictions with a score above their respective threshold are deemed as good candidates according to humans every time.

Affordances by nature are elusive to ground-truth without subjective judgement of the likelihood of an interaction. In our work, affordance location predictions are made by setting a threshold to the output (score) of the algorithm. In order to determine the threshold that produces the best results we use Amazon Mechanical Turk; where we ask people to evaluate the predictions made with our algorithms based on the smallest cell sizes. A total of 4.8 K example predictions were shown to 69 humans evaluators (turkers). These subjects had to select a “winner” from two possible options showing affordance predictions made with different scores; for instance, option 1 would show *Placing*-bowl with score of 0.75 and option 2 would show *Placing*-bowl with score 0.6. Using these pairwise human judgements, we fit a Bradley-Terry model Bradley and Terry (1952) to compute the “true” ranking of human evaluations; with this ranking we assess the performance of our algorithm. Figure 7 shows the family of classifiers induced by setting different threshold values at the score of the iT agglomeration and saliency-based iT algorithms. In this figure it can be seen that both methods achieved good performance according to human criteria yet the optimal thresholds are different. The method based on agglomeration and clustering of iT descriptors needs a threshold at 0.7 in order to produce a prediction that agrees with human criteria; on the other hand, the saliency-based method performs similarly with a threshold at 0.5. This is related to the fact that, as seen in the following subsection, our saliency-based method achieves higher precision rates; meaning that we can relax the threshold without compromising the quality of the predictions.

#### 5.3.2. Individual vs. Multiple Predictions

By now we have demonstrated that the predictions made with our algorithm align well with what humans expect to afford in a given scene/location. In an effort to further assess the performance of our algorithms we compare our multiple affordance predictions against those produced in a single-affordance scenario.

These “baseline” predictions are obtained by individually testing all affordances using the single-affordance prediction algorithm, as in Ruiz and Mayol-Cuevas (2018), for every affordance-object pair in our study. We treat these as ground-truth in order to compute performance metrics for our predictions.

For comparison, we include two additional versions of our algorithms. In the case of iT agglomerations, we tested keeping all the keypoints (and their associated provenance vectors) inside the cell during clustering. This is shown as “iT-All” and the intention of this was to investigate the possible loss of information caused by only considering the closest per-affordance keypoint inside each cell. For the case of our saliency-based method, we consider an alternative approach where salient locations are learned individually per affordance and then their corresponding keypoints are agglomerated to produce a multiple-affordance representation. This saliency-based alternative is shown as “Single.” We also show the performance achieved by the modified PointNet++ architecture used to learn saliency (shown as m-PointNet++) when tested on its own. Table 1 shows the average performance of the methods being investigated, where it can be observed that the saliency-based method showed overall better performance.

**Table 1:** Average performance of the methods for multiple affordance detection in terms of Area Under the PR Curve (AUC).

**Method**	**Cell-size [cm]**	**AUC**
iT Agglomeration + Saliency	0.5	**0.6816**
	1	0.4588
	Single	0.2722
iT Agglomeration	0.5	0.5467
	1	0.3043
	iT-All	0.3102
m-PointNet++		0.1879

*Our proposed combination of geometry and deep saliency (iT Agglomeration+Saliencey), outperforms the solely geometric (iT Agglomeration), and the solely deep learning (m-PointNet++) methods. Highlighted value shows our best performing method*.

**Figure 8:**
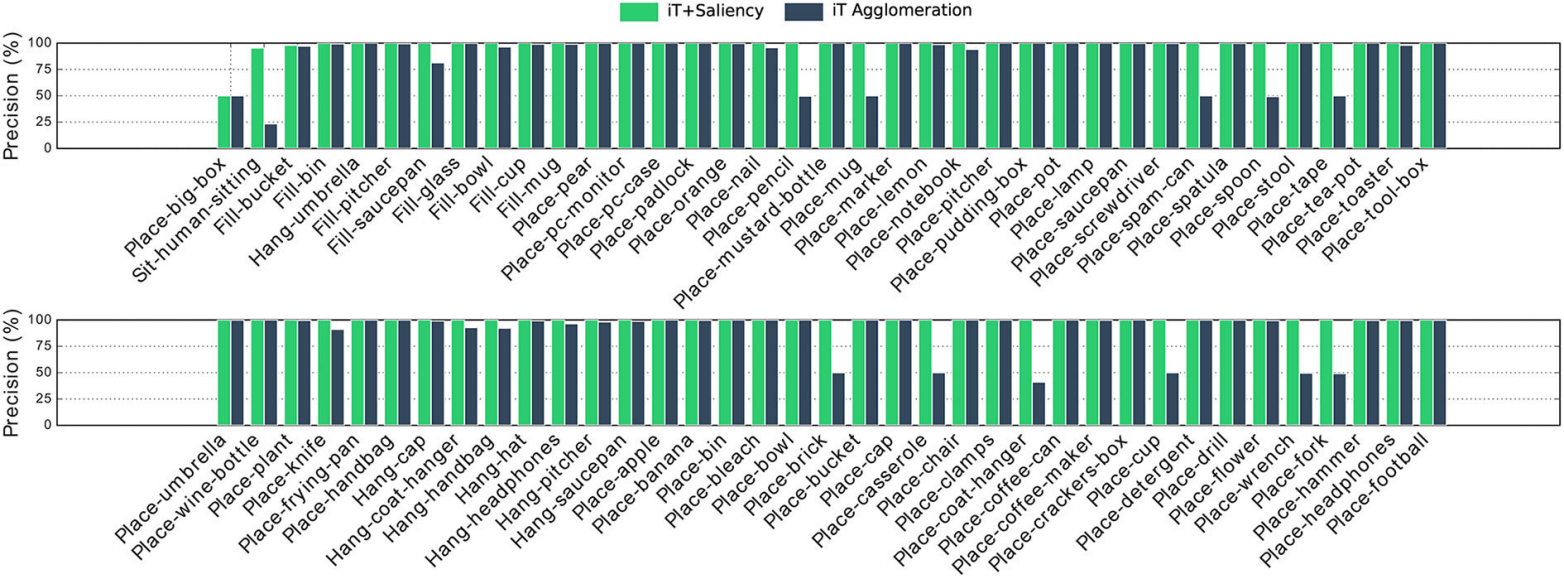
Precision achieved with multiple-affordance representations of cell size 0.5 cm. Important differences are noted in *Placing* affordances which were largely regarded as the *easiest* interactions. Interestingly both approaches struggle the most with *Placing big-box*, which has several short-length vertical provenance vectors (under the box) difficult to match during testing.

**Figure 9:**
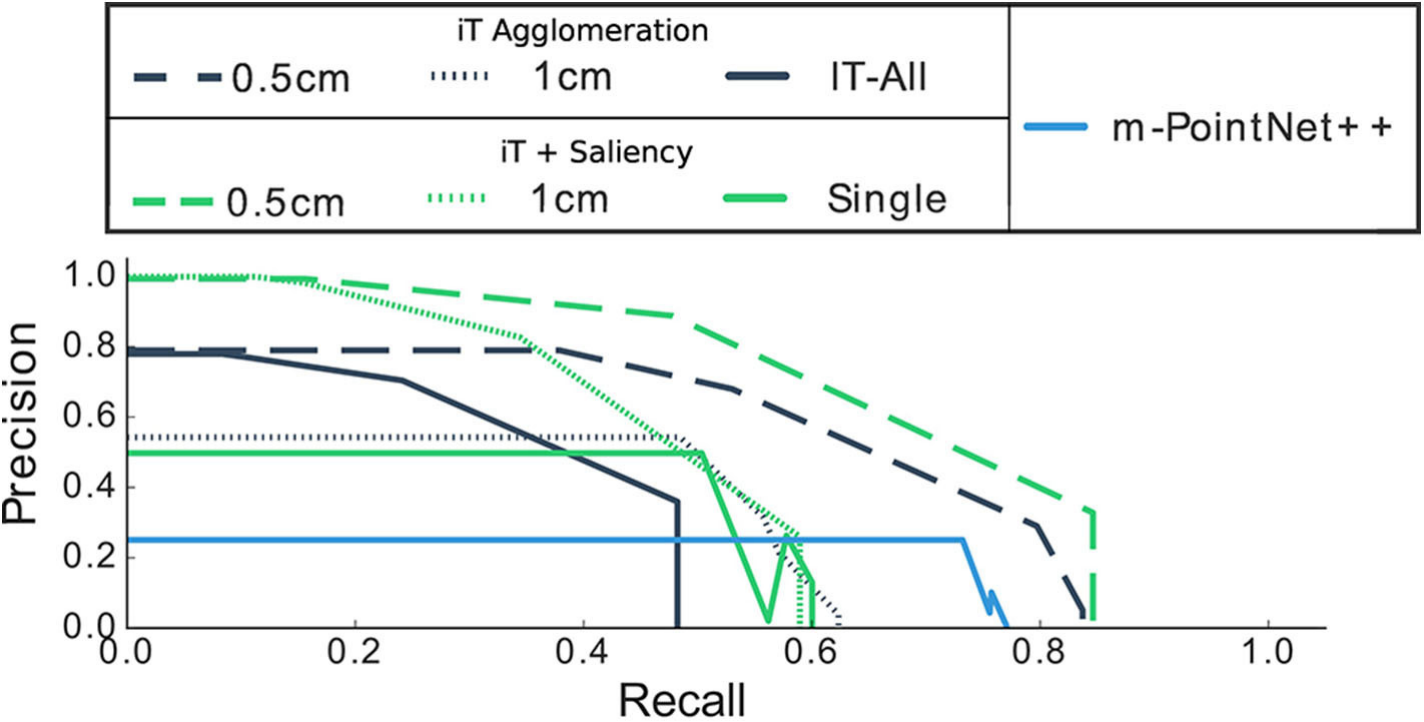
Precision-Recall curves of our methods when compared with predictions made by the single-affordance algorithm. Observe that precision drops to zero at specific recall values, this is associated with the quantization error introduced by cell clustering.

It should be noted that there is an important imbalance in affordance data. For instance, consider a kitchen environment for *Filling* affordances; there is usually one location that truly affords these interactions: the faucet/tap. In this scenario, almost every single location is a negative example for this affordance, by always predicting “background” we could achieve very high accuracy. For this reason, we evaluate with precision-recall metrics, Figures 8, 9 show the precision and recall values achieved with our algorithms.

It is worth mentioning that the iT agglomeration method performed favorably even when a single *training* example is considered. Then, by combining the interaction tensors with the salient locations learned by the network we are able to improve the performance and, as shall be shown later, more quickly assess the interaction opportunities of a given location in the scene. The deep network on its own denoted a rather poor performance; when compared with single affordance predictions the network was outperformed by all other methods. We think this highlights the importance of the geometric features that the iT is able to describe. Figure 8 shows per-affordance precision achieved by our top performing methods.

From Figure 9, it is worth noting that none of our methods achieves 100% recall, this means that we are not able to recover or predict every possible affordance in every location. This is the compromise we make in order to perform fast and multiple-affordance predictions. In other words, the methods here presented perform well if the task is to quickly evaluate the affordance possibilities at any given location with high precision; but, if the task is to retrieve all possible “combinations” or every affordance that exists across all the scene while speed is not crucial, performing single-affordance predictions is perhaps a better approach. In spite of this, we show next that our predictions are equivalent to those produced by the single affordance predictions.

##### 5.3.2.1. Human evaluation

We assess the predictions made with our algorithms by asking human evaluators to select from two options the one that best depicted the intended interactions. These options consisted on: the top-1 predictions made by the single affordance algorithm and the top-1 predictions of our multiple-affordance method, shown in a per-affordance basis. Additionally, among the options shown to people we included top prediction made with a *naive* baseline method that uses Iterative Closest Point (ICP)^1^. This baseline computes a score from the best alignment (i.e., rigid transformation) between a target pointcloud (interaction training example) and the pointcloud being tested. A total of 1,200 pair-wise comparisons were shown to 48 turkers. We found that 48% of the time people chose the multiple-affordance predictions as the best option when compared against single-affordance predictions. Note that a random guess is 50%, which means that our predictions are regarded as good as the single affordance prediction “ground truth.” On the other hand, when compared with the ICP baseline, our predictions were chosen 87% of the time.

#### 5.3.3. Frame Rates and Quantization

**Figure 10:**
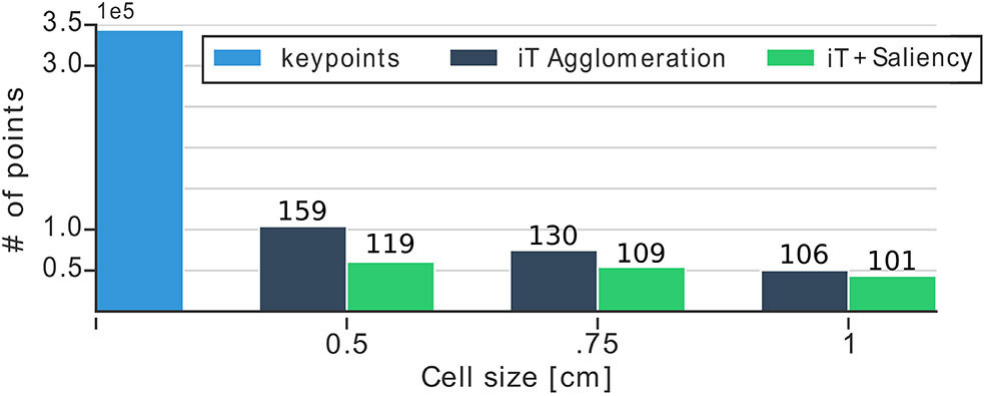
Bar plot shows the dimensionality reduction achieved with our methods for different cell sizes. We reduce up to six times the number of keypoints required to make predictions. Numbers above each bar show prediction time (milliseconds) per test-point of the input scene. For instance, with the representation with 1 cm cells, we are able to predict up to 84 affordances in any scene location in 101 ms.

**Figure 11:**
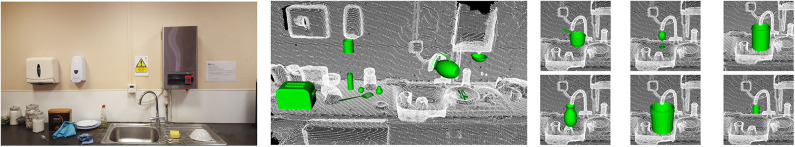
Multiple interactions afforded by the same pointcloud predicted in a never seen before scene. The predictions shown in this figure are carried out simultaneously and at high frame rates with our approach. Note the tap in the kitchen which, in addition to afford *filling* a variety of objects, it also affords *hanging*; both interaction possibilities rightly predicted by our method.

We evaluate the effect that the size of cells in the grid has in terms of speed or prediction time for the methods we propose. Figure 10 shows the dimensionality of our multiple-affordance representation and the prediction rates according to the cell size. Looking at this figure it stands out the large reduction that we are able to achieve with our proposed approaches: both of them reduce the number of points in the representation by nearly 6 times (344 vs. 60 K keypoints). The prediction rates on the same figure show that using grids with a cell size of 1 cm^3^ allows us to detect up to 84 affordances at 10 different locations per iteration on the input scene. This is significantly faster (7x improvement) than predicting affordances by trying 84 descriptors at test time, which would require 840 ms per test-point (average of 10 ms per affordance as reported in Ruiz and Mayol-Cuevas, 2018). Due to the fact that our prediction algorithm performs a NN-search in order to estimate test-vectors and compare them against provenance vectors, the complexity of such operation depends heavily on the dimension of the multiple-affordance representation (i.e., the number of centroids/keypoints). More points in the representation require more computations; therefore, reducing the representation allows us to perform faster evaluations at test-time. Even with such a reduction in dimensionality our method, as shown earlier, is able to produce top-quality affordance predictions. Figure 11 offers further examples of our predictions that show generality and multiple affordance estimation per scene. Particularly, this figure exemplifies a common and difficult scenario for affordance prediction: multiple interactions afforded by the same scene or object; for instance, the tap in the kitchen which not only affords *filling* multiple objects but also affords *hanging*. Note that our approach deals well with this type of scenario, predicting multiple affordances, simultaneously and a high framerates.

It is worth mentioning that we homed in the strategy for uniform-size grid clustering given its straightforward implementation and performance against other alternatives explored during the early stages of our research. For instance, one alternative that we considered to devise a multiple-affordance representation investigated the sampling of keypoints from the agglomeration instead of taking into account all affordance keypoints for clustering; however, sampling had the adverse effect of drastically reducing the keypoints per affordance (i.e., under representation) in the final descriptor, which did not allow for scalability in the number and type of affordances in the representation. Another approach that we explored consisted in fitting an Octree structure to the agglomeration of affordance keypoints, which was aimed for a more *efficient* representation. However, due to the variance in sparsity and size of the affordances that we study, deciding on where to place the leaf centroids was not a straight-forward process. After all, these centroids would be employed during the NN-search used for prediction. An Octree representation would require additional steps (e.g., tree search) during prediction in novel pointclouds. These alternative explorations while not exhaustive in the space of their hyper-parameters did not indicate improvements over our current formulation. Distinct methods could provide other performance compromises, but our straightforward agglomeration will serve as a baseline for future investigations.

## 6. Conclusions and Future Work

In this work, we developed and evaluated a scalable, real-time approach for multiple geometric affordance prediction. Our approach leverages advantages of the interaction tensor, a compact geometric representation with that of scene saliency provided by a deep learning architecture. We predict up to 84 affordances on over 150 real previously unseen scenes and in a way that aligns well within the intrinsically subjective nature of affordances as validated with crowd-sourced human judgement. In such evaluation, our affordance proposals are preferred 87% of the time over other geometric baseline methods. Furthermore, we achieve four times better performance over a deep-learning-only baseline and seven times faster predictions when compared to previous art.

The current approach uses only geometry to compute the likelihood of a location in the scene of affording an interaction. This assumes that the sensor is able to perceive the scene correctly; errors in the sensing could affect the performance or quality of the predictions. One important avenue to future work is the integration of our system with more robust ways to sense the scene; for instance, methods to correctly perceive reflective surfaces or outdoors environments, where RGB-D sensors would fail. Examples of such methods are Wang et al. (2018) or Ma and Karaman (2018) for real-time monocular dense reconstructions. Another interesting direction for future work is *grasping* affordances. Currently, our method would assume that the query-object is already in the agent's hand; thus, the goal would be to detect where the agent could *Place, Hang* or *Fill* this object; future work could investigate grasping as the interaction between a hand and other objects, e.g., where robot's hand serves as a query-object that interacts with a scene-object in the environment. Our approach enables the desirable property of working from a single example to generalize to unknown scenes. However a further avenue of work should include strategies for the discovery of new affordances that can then be generalized to new interaction pairs.

Overall, affordance perception is a fundamental ability for agents that need to interact with their environment, or more generally, understand the interactions that take place (or could take place) in their surroundings. Perceiving the world in this way can lay the basis to learn more complex concepts. As a result of our affordance detection rates, we see many avenues for applications of our method; applications such as semantic scene understanding, autonomous robotics, navigation planning and augmented reality where scenes can be augmented using discovered affordances rather than pre-scripted.

## Data Availability Statement

The data and code for this research is publicly available at https://github.com/eduard626/interaction-tensor-affordances.

## Author Contributions

All the authors participated in writing the paper, conceived the experiments, and analyzed the data.

## Conflict of Interest

The authors declare that the research was conducted in the absence of any commercial or financial relationships that could be construed as a potential conflict of interest.

## Footnotes

^1^implementation from Rusu and Cousins (2011).
